# The role of chest CT in management of asymptomatic SARS-CoV-2 infections: A longitudinal multi-center study in Chongqing, China

**DOI:** 10.7150/ijms.50705

**Published:** 2021-01-01

**Authors:** Ting Chen, Dajing Guo, Jianguo Lu, Bo Xiang, Xiang Li, Jianghua Nie, Xiaojing He, Kunhua Li

**Affiliations:** 1 Department of Radiology, the Second Affiliated Hospital of Chongqing Medical University, Chongqing, China.; 2 Department of Radiology, the Second Affiliated Hospital of Chongqing Medical University at Fengjie, Chongqing, China.; 3 Department of Radiology, Yongchuan Hospital of Chongqing Medical University, Chongqing, China.; 4 Department of Radiology, Chongqing Three Gorges Central Hospital, Chongqing, China.; 5 Department of Radiology, People's Hospital of Changshou Chongqing, Chongqing, China.

**Keywords:** SARS-CoV-2, COVID-19, Asymptomatic infections, Chest CT

## Abstract

**Background:** Multiple societies including the Fleischner Society do not recommend that CT is routinely used in asymptomatic SARS-CoV-2 infections; however, this advice is based on the limited evidence. In this study, we aim to confirm whether it is necessary to do CT scans in SARS-CoV-2 asymptomatic infections by summarizing the longitudinal chest CT and clinical features of asymptomatic SARS-CoV-2 infections.

**Methods:** A total of 33 individuals (14 men and 19 women) with asymptomatic SARS-CoV-2 infections were retrospectively enrolled. Clinical data of CT positive and negative groups were compared. Longitudinal chest CT scans were reviewed for CT features and analyzed for temporal change.

**Results:** Thirty-two (97%) individuals had positive results for first RT-PCR testing. For clinical data, only monocyte count showed a significant difference between CT positive and negative groups. For first chest CT, only eighteen (54.5%) individuals had abnormal manifestations, common CT features were GGO (88.9%) and consolidation (33.3%), the median number of segments involved was 3.0 (1.0-7.5). No case in CT negative group was abnormal on the follow-up CT. Three patterns of evolution throughout series of CT were observed in CT positive group, including gradual improvement (12, 66.7%), mismatch to improvement (3, 16.7%) and mild progression to improvement (3, 16.7%). On last CT scans, most cases had radiographic improvement but residual abnormalities. Significant differences were exhibited in density, long diameter, number of lung segments involved, and percentage of consolidation between the first and last CT scans. All cases had stable conditions and finally confirmed negative for SARS-CoV-2 RT-PCR tests without developing into severe pneumonia.

**Conclusion:** Considering poor performance of CT in screening, stable conditions during followup, and good outcomes in asymptomatic SARS-CoV-2 infections, we confirm that it is unnecessary to do CT scans in asymptomatic SARS-CoV-2 infections.

## Introduction

In December 2019, a novel coronavirus, severe acute respiratory syndrome coronavirus 2 (SARS-CoV-2) causing human disease officially named Corona Virus Disease 2019 (COVID-19), was found in Wuhan, Hubei Province, China 1-5. Currently, human-to-human transmission of the virus accounts for most infections worldwide 6. And this disease has spread to an increasing number of countries, areas or territories around the globe; there were more confirmed cases reported outside China than inside China, and many new epicentres of spread have emerged 7. On the basis of “alarming levels of spread and severity, and by the alarming levels of inaction”, on March 11, 2020, the COVID-19 situation was characterized as a pandemic by the Director-General of WHO 8,9. Up to 2 November 2020, WHO has officially reported over 46 million confirmed COVID-19 cases and 1.1 million confirmed deaths 7.

Asymptomatic SARS-CoV-2 infections have been evaluated to comprise 18%-46% of all infections 10-12. As asymptomatic infections are very covert and may be a vital contagious source 13-19. Besides early studies showed low sensitivity of SARS-CoV-2 reverse transcriptase-polymerase chain reaction (RT-PCR) test (the golden standard for confirmation of SARS-CoV-2 infection) and high sensitivity of chest CT 20-23. So numerous studies recommend that combining assessment of chest CT and RT-PCR could facilitate early diagnosis of SARS-CoV-2 infections 20, 21, 24-26, consequently, the number of CTs performed in persons under investigation for SARS-CoV-2 infections has increased 27. However, according to recent statements of multiple societies including the Fleischner Society 28, CT is not routinely indicated as a screening test for COVID-19 in asymptomatic individuals, based on the limited evidence. For this reason, we summarized the longitudinal chest CT and clinical features of asymptomatic SARS-CoV-2 infections to confirm whether it is necessary to do CT scans in asymptomatic infections.

## Materials and Methods

This retrospective study was approved by the Institutional Ethics Committee of the Second Affiliated Hospital of Chongqing Medical University, and the requirement for informed consent was waived.

### Study population

According to the Prevention and Control of COVID-19 of China (version sixth) 29, all individuals confirmed as asymptomatic SARS-CoV-2 infections by RT-PCR from January 2020 to February 2020 were recruited in this study. The inclusion criteria were as follows: 1) all cases were asymptomatic when they were confirmed as SARS-CoV-2 infections and were subsequently hospitalized isolation; 2) all cases underwent first chest CT (obtained within two days of first positive SARS-CoV-2 result) and last CT (obtained within two days of first negative SARS-CoV-2 result from two consecutive detection) examinations; 3) all cases were followed up to discharge. The exclusion criteria: having severe artifacts on their CT images. Finally, a total of 33 individuals (14 men and 19 women) were included in the study with mean age of 43.2 years (SD 14.3), including 10 individuals from the Second Affiliated Hospital of Chongqing Medical University at Fengjie, 10 individuals from Yongchuan Hospital of Chongqing Medical University, nine individuals from Chongqing Three Gorges Central Hospital, and four individuals from People's Hospital of Changshou Chongqing.

According to the first chest CT manifestations, the eligible individuals were split into two groups: CT positive and negative groups. The clinical parameters included age, gender, signs, laboratory findings, and time of PT-PCR conversion (calculated from the day when SARS-CoV-2 was detected by RT-PCR to the first day of two consecutive negative results of RT-PCR) were collected and evaluated.

### CT examinations and imaging evaluation

All chest CT scans were obtained using four multi-detector CT scanners: SOMATOM go.Top (Siemens Healthineers, Germany), SOMATOM Sensation 16 (Siemens Healthineers, Germany), Light Speed16 (GE Medical Systems, USA) and Asteion (TOSHIBA, Japan). Two chest radiologists with 10 and 8 years of experience who were blinded to the clinical data evaluated the CT findings in consensus. For each of the individuals, the first and last chest CT images were evaluated for the following characteristics based on the Fleischner Society Nomenclature recommendations 30 and similar studies 24,31: ground-glass opacity (GGO), consolidation, linear opacities, interlobular septal thickening, crazy-paving pattern, “spider web sign”, subpleural curvilinear line, thickening of the adjacent pleura, lymphadenopathy, pleural effusion and pericardial effusion. What's more, the margin definition of the max lesion, distribution, location and extent of abnormalities were recorded 24.

All the follow-up CT images were evaluated for: 1) the patterns of evolution throughout the series of CT scans 24, 2) the long diameter (cm) and density (HU) of the max lesion of the lung, 3) the number of segments involved.

The median volume CT dose index and dose-length product for CT acquisition were 11.1 mGy (range, 5.5-18.4) and 364.8 mGy∙cm (range, 215-750), respectively, corresponding to an effective radiation dose of 5.1 mSv (range, 3.0-10.5) (using a standard conversion factor for chest CT of 0.014 mSv/mGy∙cm).

### Statistical Analysis

Day 0 was defined as the day of first positive SARS-CoV-2 result. Categorical variables were expressed as number (%), and quantitative variables were expressed as mean (SD) or median (interquartile range, IQR) values. Comparisons of clinical features between CT positive and CT negative groups, χ^2^ test and Fisher exact test were used for categorical variables. Quantitative variables were tested for normality by using Shapiro-Wilk tests, normally distributed data were analyzed by independent sample *t* test; otherwise, the Mann-Whitney U test was used. Comparisons of CT features between first and last CT scans were performed by using paired Student *t* test or Wilcoxon sign-rank test for continuous data and the McNemar test or Marginal Homogeneity test for categorical data. Differences with *p* < 0.05 were considered statistically significant. All statistical analyses were done by using SPSS statistical software (version 20.0 IBM).

## Results

### Subject characteristics

All individuals had some contact with SARS-CoV-2 infections. Thirty-two (97%) individuals had positive results for first RT-PCR testing, one individual had positive results for second RT-PCR testing. Multiple laboratory indicators were abnormal; the common features were increased procalcitonin (57.6%) and decreased lymphocyte count (36.4%). Individuals were assigned to two groups on the basis of CT findings: CT positive group (18/33, 54.5%) and CT negative group (15/33, 45.5%). The clinical characteristics and laboratory results of individuals by group were summarized in the **Table 1.** No significant differences in age (*p* = 0.276) or sex distribution (*p* = 0.062) between groups were identified. For all the signs of admission, no significant differences were found between the two groups (*p >* 0.05). The monocyte count of CT positive group was significantly higher compared with that of CT negative group; however the other laboratory parameters were not significantly different for the two groups. More details were summarized in the **Table 1.**

During the follow up, although five individuals (27.8%) in CT positive group and four individuals (26.7%) in CT negative group occurred mild symptoms after an average of three days from first CT scans, all individuals had stable conditions and finally confirmed negative for SARS-CoV-2 RT-PCR tests without developing into severe pneumonia. The time for RT-PCR conversion of CT positive group and CT negative group was 13 days (IQR, 8-9) and 11 days (IQR, 6-14), respectively; and there was no statistical difference between the two groups.

### First chest CT findings

Eighteen (54.5%) individuals had abnormal CT manifestations of infections. The common CT features included GGO (16/18, 88.9%) and consolidation (6/18, 33.3%) (**Table 2**). Two (11.1%) individuals had linear opacities, and one (5.6%) individual had subpleural curvilinear line. Lymphadenopathy, pleural effusion and pericardial effusion were absent in all individuals. Max lesions of 12 (66.7%) individuals were ill-defined margins. The median long diameter and density of the max lesion were 2.3 cm and -305.6HU, respectively.

The common lung segments involved were lateral basal segments of bilateral lower lobes and posterior segment of right lower lobe. The median number of segments involved was 3.0 (IQR, 1.0-7.5). Ten (55.6%) individuals had bilateral lung involvement, 12 (66.7%) individuals showed subpleural distribution, 12 (66.7%) individuals showed posterior distribution, and eight (44.4%) showed diffuse distribution of CT abnormalities.

### Follow-up chest CT findings

No case in CT negative group was abnormal on the follow-up CT. Eighteen individuals of CT positive group had at least one follow-up chest CT examination. The mean interval time from first CT to last CT was 12.4 days (SD, 6.6). Three patterns of evolution throughout the series of CT scans were observed among these 18 individuals: gradual improvement (type-GI), followed by mismatch to improvement (type-MI), and mild progression to improvement (type-PI).

**Figure 1:**
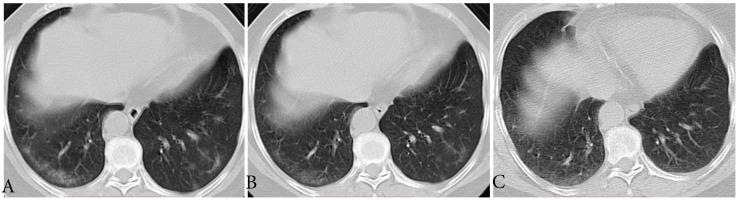
** Typical evolution of type-GI in a 61-year-old female with asymptomatic SARS-CoV-2 infection. A,** Day 0, the first chest CT showed multifocal lesions of subpleural GGO in bilateral lower lobe. **B,** Day 3, obvious resolution of the first GGO was observed. **C,** Day 6, continued resolution with minimal residual GGO was observed, the patient had two consecutive negative results of RT-PCR (day 6 and 7). GGO, ground-glass opacity; GI, gradual improvement; SARS-CoV-2, severe acute respiratory syndrome coronavirus 2.

The CT findings of 12 (66.7%) individuals showed type-GI, that is decrease in extent and density from the first to last CT scans (**Fig. 1**). The median interval time from first CT scans to last CT scans was 7.5 days (range, 3-18). Three of them had mild coughs, and the period from the date of first CT scans to the symptom onset ranged from 2 to 3 days. All of the 12 individuals had RT-PCR conversion with a median interval of 9.5 days (range, 5-19).

**Figure 2:**
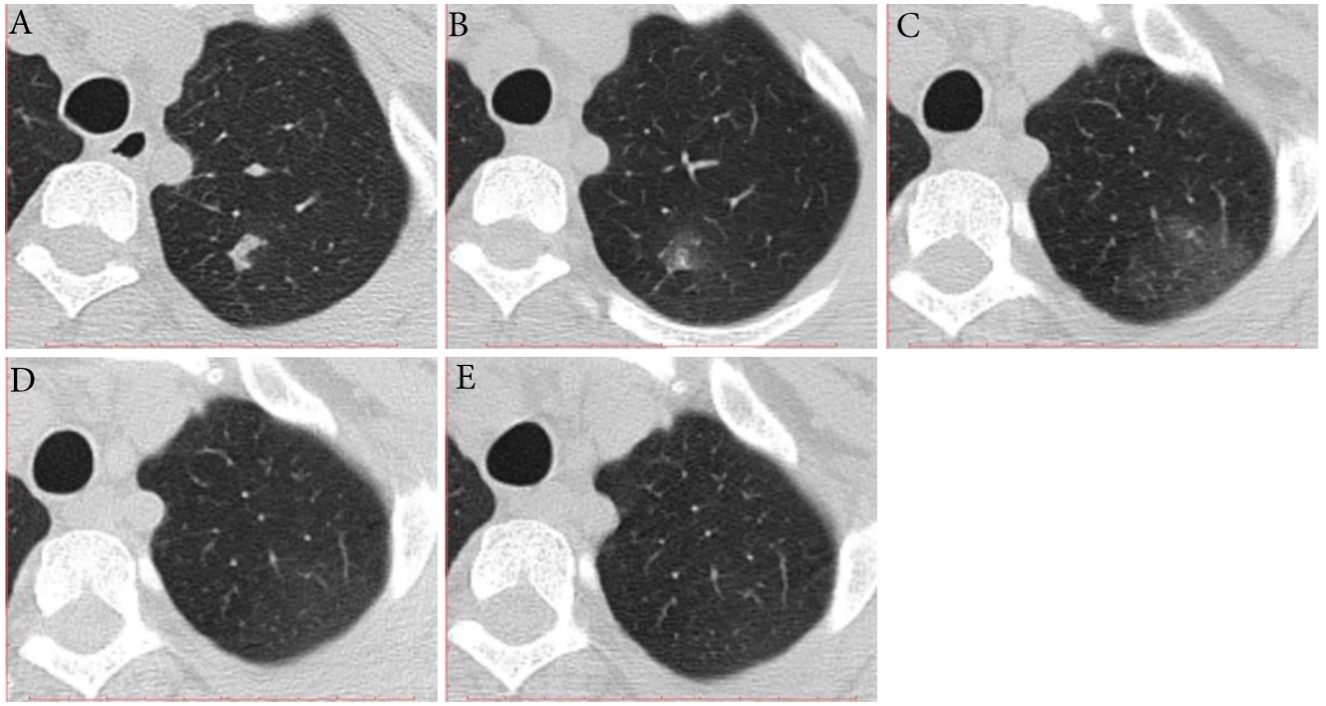
** Typical evolution of type-MI in a 39-year-old female with asymptomatic SARS-CoV-2 infection. A,** Day 0, the first chest CT showed a focal consolidation in apical posterior segment of left upper lobe. **B-C,** Day 2 and 6, the second to third CT scans showed increase in extent and decrease in density. **D,** Day 10, obvious resolution of the previous GGO was observed. **E,** Day 14, full resolution of the lesion was observed; and day 14 and 15, the patient had two consecutive negative results of RT-PCR. GGO, ground-glass opacity; MI, mismatch to improvement; RT-PCR, reverse transcriptase-polymerase chain reaction.

Three (16.7%) individuals showed type-MI, that is the trends of the extent and density were not similar to each other in the early stage and then improvement (**Fig. 2**). Two individuals showed the increase in long diameter and decrease in density on the second CT scans (2-3 days after the first positive SARS-CoV-2 result), followed by improvement. What's more, one individual showed the decrease in long diameter and increase in density on the second CT scan (7 days after the first positive SARS-CoV-2 result), followed by improvement. The interval time from first CT scans to last CT scans ranged from 14 to 24 days. The time for RT-PCR conversion of the three individuals ranged 14 to 26 days.

**Figure 3:**
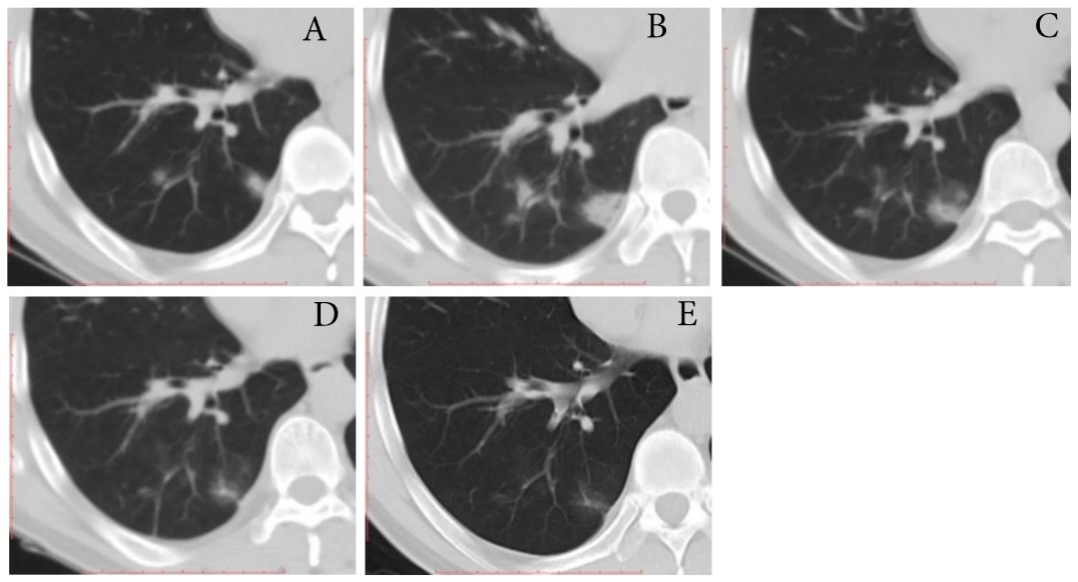
** Typical evolution of type-PI in a 48-year-old male with asymptomatic SARS-CoV-2 infection. A,** Day 0, the first chest CT showed multifocal lesions of consolidation in right lower lobe. **B,** Day 2, mild progression was showed on the second CT scan. **C-E,** Day 8-21, gradual resolution was observed on the third to last CT scans, and minimal residual GGO and linear opacities were observed on the last CT scan; And day 21 and 22, the patient had two consecutive negative results of RT-PCR. GGO, ground-glass opacity; PI, progression to improvement; RT-PCR, reverse transcriptase-polymerase chain reaction.

Three (16.7%) individuals showed type-PI, that is the mild increase in extent and density on the second CT scans (3 days [range, 2-4 days] after first positive SARS-CoV-2 result) and then improvement (**Fig. 3**). The interval time from first CT scans to last CT scans ranged from 21 to 23 days. One individual had a mild cough at the time of progress. The time for RT-PCR conversion of the three individuals ranged 19 to 21 days.

### Last chest CT findings

On the last CT scans, two individuals had complete resolution of lung abnormalities, and the other 16 individuals had residual lesions including GGO (88.9%) and linear opacities (22.2%). The percentage of consolidation showed a decrease on the first CT to last CT, and the difference was statistically significant (*p* = 0.031). The median long diameter and density of the max lesion on the last CT were 1.7cm and -701.9HU, respectively, which were statistically smaller than those of the first CT (**Table 2**).

The median number of segments involved on last CT (1.0 [IQR, 1.0-3.2]) was significantly smaller than that of the first CT (3.0 [IQR, 1.0-7.5], *p* = 0.005). The percent of bilateral distribution, random distribution, both anterior and posterior distribution, and diffuse involvement showed decreased on the first CT to last CT (**Table 3**).

## Discussion

Although the literature on SARS-CoV-2 infections has grown exponentially, most of them focused on symptomatic patients 24,32-35, especially on severe and critical patients 31,35,36. Recently, several studies have reported chest CT of asymptomatic SARS-CoV-2 infections. However, most of these asymptomatic studies were cross-sectional and focused on summarizing of CT features or screening 25,37-39. A comprehensive and longitudinal study (including screening, CT features and evolution of asymptomatic SARS-CoV-2 infections) was scarcely reported. Besides, the chest CT role in early studies may be overestimated and the overuse of CT will inevitably increase the risk of radiation damage and cross-infection. So multiple societies including the Fleischner Society do not recommend that CT is routinely used in asymptomatic individuals 28, however, this advice is based on the limited evidence. In this context, we conducted this study to confirm whether it is necessary to do CT scans of screening and follow-up in asymptomatic infections. We found low sensitivity of initial CT, no obvious progression of lung lesions and stable conditions during followup, and good outcomes in asymptomatic SARS-CoV-2 infections. And we found a novel pattern (type-MI) of evolution in asymptomatic SARS-CoV-2 infections.

In this study, only eighteen (54.5%) of asymptomatic SARS-CoV-2 infections had abnormal chest CT findings, which indicates that normal chest CT cannot exclude the diagnosis of SARS-CoV-2 infection. This is in line with low sensitivity of chest CT for asymptomatic SARS-CoV-2 infections from the cruise ship “Diamond Princess” 40. The superior sensitivity in earlier literature was likely biased toward symptomatic patients imaged in later stages of disease 41. The most common abnormal CT feature in the asymptomatic SARS-CoV-2 infections was GGO with predominantly bilateral, subpleural and diffuse involvement, which was similar to those of the symptomatic SARS-CoV-2 infections 24,31. But the median number of segments involved in our study was less than that of symptomatic infections 24,31 and the consolidation in the asymptomatic SARS-CoV-2 infections was focal, which suggests that lung involvements of asymptomatic SARS-CoV-2 infections are less extensive than those of symptomatic SARS-CoV-2 infections. Although the CT findings of asymptomatic SARS-CoV-2 infections were characteristic, the sensitivity of CT was only modest and definite diagnosis still requires a positive RT-PCR test, this suggests limited value of chest CT as a screening test. Fortunately, the sensitivity of initial RT-PCR test reached up to 97% in our study. While early studies of test performance in Wuhan showed significantly lower sensitivities 21-23, this could be explained by different enrollment criteria, sample sizes, testing capacities, kit performances, and stages of infections 28. Even in this scenario, multiple RT-PCR testing should be the first choice to exclude the diagnosis if no constraint on RT-PCR testing exists 28,42. And a recent study 43 found that antibody testing may provide additional value on identification of asymptomatic infections with negative RT-PCR results.

Compared with the CT negative group, only monocyte count was observed significantly higher in the CT positive group. This could be explained by compensation to infiltration of pulmonary monocytes 44. The other indicators especially including time of RT-PCR conversion showed no statistical differences between CT positive and negative groups. This suggests that initial CT can provide little additional value in clinical practice for asymptomatic SARS-CoV-2 infections.

No case in CT negative group was abnormal on the follow-up CT. Three patterns of evolution throughout the longitudinal CT scans were observed among these 18 CT positive individuals: type-GI, followed by type-MI, and type-PI. Noticeably, the type-MI was a novel pattern of evolution that we found in asymptomatic SARS-CoV-2 infections. The type-MI infers that progression and improvement occur simultaneously during the mismatch stage from different dimensions. What's more, we found that asymptomatic SARS-CoV-2 infections had lower incidence and milder progression of type-PI compared with symptomatic SARS-CoV-2 infections of previous studies 24,32,33. In addition, for type-MI or type-PI in our study, the persistence of high levels for lung lesions in asymptomatic SARS-CoV-2 infections was not observed, while it was very often seen in symptomatic SARS-CoV-2 infections; after that, the asymptomatic SARS-CoV-2 infections in our study showed a faster decrease in lung lesions 32,34. Besides all cases in our study had stable conditions and finally confirmed negative for SARS-CoV-2 RT-PCR testing without developing into severe pneumonia. Thus, we can infer that the asymptomatic SARS-CoV-2 infections have less severity and more favourable outcomes than those of symptomatic SARS-CoV-2 infections. So we suggest that a different strategy with no need for CT follow-up in asymptomatic individuals should be taken for avoiding unnecessary radiation damage and reducing the risk of cross-infection, compared with the symptomatic patients.

Noticeably, although the individuals in our study already confirmed negative for RT-PCR before discharge from isolation, the last CT of most cases still showed abnormalities mainly including GGO. A previous study 45 found that some recovered individuals who met criteria for hospital discharge had positive results for SARS-CoV-2 infections five to 13 days later. A recent pathological examination confirmed SARS-CoV-2-viruses remaining in pneumocytes and virus-caused pathological changes in the lungs of a ready-for-discharge patient 46. This indicates that self-monitoring of health status, isolation at home, and further follow-up including RT-PCR testing will be required after discharge.

This study has several limitations. Firstly, the sample size of the asymptomatic SARS-CoV-2 infections was relatively small. Statistical tests and *p* values should be interpreted with caution because of the small sample size. Secondly, because SARS-CoV-2 infection is a sudden novel emergency and highly contagious, in the early stage with no experience for reference, chest CT followup scans of some individuals were frequent in our study. Thirdly, the CT scans for the included patients might have different time intervals from the date of being infected.

In conclusion, considering poor performance of CT in screening, no obvious progression of lung lesions and stable conditions during followup, and good outcomes in asymptomatic SARS-CoV-2 infections, we confirm that it is unnecessary to do CT scans in asymptomatic infections.

## Figures and Tables

**Table 1:** Clinical characteristics and laboratory findings of asymptomatic SARS-CoV-2 infections

Parameter	Total (n=33)	CT positive (n=18)	CT negative (n=15)	*p*
Age (y)	43.2 (14.3)	45.7 (15.0)	40.2 (13.3)	0.276
**Sex**				0.062
Male	14 (42.4%)	5 (27.8%)	9 (60.0%)	
Female	19 (57.6%)	13 (72.2%)	6 (40.0%)	
Temperature (°C)	36.50 (36.30-36.60)	36.50 (36.20-36.60)	36.40 (36.40-36.60)	0.929
Heart rate (bpm)	79.58 (8.23)	81.50 (8.75)	77.27 (7.17)	0.144
Respiratory rate	20.00 (19.50-20.00)	20.00 (19.75-20.25)	20.00 (19.00-20.00)	0.421
**White blood cell count (×10^9^/L)**	6.35 (5.24-7.02)	6.11 (5.25-6.64)	6.90 (5.18-7.47)	0.343
Increased	3 (9.1%)	2 (11.1%)	1 (6.7%)	1.000
Decreased	1 (3.0%)	0 (0.0%)	1 (6.7%)	0.455
**Neutrophil ratio (%)**	65.20 (60.25-75.75)	62.60 (59.23-76.00)	69.80 (63.70-75.60)	0.274
Increased	10 (30.3%)	6 (33.3%)	4 (26.7%)	0.927
**Lymphocyte ratio (%)**	23.68 (9.21)	22.64 (9.83)	24.93 (8.57)	0.487
Decreased	10 (30.3%)	7 (38.9%)	3 (20.0%)	0.426
**Monocyte ratio (%)**	7.07 (3.09)	7.99 (3.46)	5.97 (2.24)	0.061
Increased	5 (15.2%)	4 (22.2%)	1 (6.7%)	0.346
Decreased	1 (3.0%)	1 (5.6%)	0 (0.0%)	1.000
**Neutrophil count (×10^9^/L)**	4.10 (2.93-4.98)	3.94 (2.90-4.86)	4.39 (3.03-5.75)	0.442
Increased	4 (12.1%)	3 (16.7%)	1 (6.7%)	0.607
Decreased	1 (3.0%)	0 (0.0%)	1 (6.7%)	0.455
**Lymphocyte count (×10^9^/L)**	1.35 (0.46)	1.34 (0.44)	1.37 (0.50)	0.878
Decreased	12 (36.4%)	7 (38.9%)	5 (33.3%)	0.741
**Monocyte count (×10^9^/L)**	0.38 (0.14)	0.43 (0.15)	0.32 (0.11)	**0.023**
Increased	2 (6.1%)	2 (11.1%)	0 (0.0%)	0.489
**Procalcitonin (ng/mL)**	0.050 (0.030-0.068)	0.050 (0.038-0.075)	0.050 (0.030-0.060)	0.682
Increased	19 (57.6%)	11 (61.1%)	8 (53.3%)	0.653
Mild symptoms on during the follow-up	9 (27.3%)	5 (27.8%)	4 (26.7%)	1.000
Time with mild symptoms onset after first CT (d)	3.0 (2.5-7.0)	3.0 (2.5-5.5)	5.5 (2.0-7.5)	0.556
Time of RT-PCR conversion (d)	12.0 (7.5-15.5)	13.0 (8.0-19.0)	11.0 (6.0-14.0)	0.215

Data are n (%), mean (SD), median (IQR). Increased means over the upper limit of the normal range and decreased means below the lower limit of the normal range. bpm, beats per minute; RT-PCR, reverse transcriptase-polymerase chain reaction; SARS-CoV-2, severe acute respiratory syndrome coronavirus 2.

**Table 2:** First and last CT features of CT positive group with asymptomatic SARS-CoV-2 infections

	First CT scan (n = 18)	Last CT scan (n = 18)	*p*
Margin definition			0.102
Well-defined	4 (22.2%)	3 (16.7%)	
Ill-defined	12 (66.7%)	12 (66.7%)	
Partial ill-defined	2 (11.1%)	1 (5.6%)	
No lesion	/	2 (11.1%)	
CT value (HU)	-305.6 ([-519.9]- [-108.1])	-701.9 ([-743.5]-[-540.3])	**0.003**
Long diameter (cm)	2.3 (1.6-4.2)	1.7 (1.2-3.1)	**0.001**
Ground glass opacity	16 (88.9%)	16 (88.9%)	1.000
Consolidation	6 (33.3%)	0 (0.0%)	**0.031**
Linear opacities	2 (11.1%)	4 (22.2%)	0.625
Interlobular septal thickening	2 (11.1%)	0 (0.0%)	0.500
Crazy-paving pattern	1 (5.6%)	0 (0.0%)	1.000
Spider web sign	4 (22.2%)	0 (0.0%)	0.125
Subpleural curvilinear line	1 (5.6%)	0 (0.0%)	1.000
Thickening of the adjacent pleura	4 (22.2%)	1 (5.6%)	0.375

Data are n (%), median (IQR). Percentages may not total 100 because of rounding. SARS-CoV-2, severe acute respiratory syndrome coronavirus 2.

**Table 3:** First and last CT distribution and extent of lung lesions of CT positive group with asymptomatic SARS-CoV-2 infections

The lung segment involved	First CT scan(n = 18)	Last CT scan(n = 18)	*p*
**Left upper lobe**			
Apical posterior	7 (38.9%)	3 (16.7%)	0.125
Anterior	3 (16.7%)	1 (5.6%)	0.500
Superior lingula	3 (16.7%)	0 (0.0%)	0.250
Inferior lingula	3 (16.7%)	0 (0.0%)	0.250
**Left lower lobe**			
Superior	5 (27.8%)	1 (5.6%)	0.125
Medial Anterior basal	5 (27.8%)	1 (5.6%)	0.125
Lateral basal	9 (50.0%)	7 (38.9%)	0.500
Posterior	4 (22.2%)	3 (16.7%)	1.000
**Right upper lobe**			
Apical	3 (16.7%)	0 (0.0%)	0.250
Posterior	5 (27.8%)	3 (16.7%)	0.500
Anterior	3 (16.7%)	1 (5.6%)	0.500
**Right middle lobe**			
Lateral	4 (22.2%)	1 (5.6%)	0.250
Medial	2 (11.1%)	0 (0.0%)	0.500
**Right lower lobe**			
Superior	5 (27.8%)	3 (16.7%)	0.500
Medial basal	0 (0.0%)	0 (0.0%)	/
Anterior	3 (16.7%)	0 (0.0%)	0.250
Lateral basal	9 (50.0%)	8 (44.4%)	1.000
Posterior	8 (44.4%)	7 (38.9%)	1.000
Number of segments involved	3.0 (1.0-7.5)	1.0 (1.0-3.2)	**0.005**
**Lung involvement**			0.102
Unilateral	8 (44.4%)	8 (44.4%)	
Bilateral	10 (55.6%)	8 (44.4%)	
No lesion	/	2 (11.1%)	
**Distribution**			0.059
Subpleural	12 (66.7%)	13 (72.2%)	
Peribronchovasular	1 (5.6%)	0 (0.0%)	
Random	5 (27.8%)	3 (16.7%)	
No lesion	/	2 (11.1%)	
**Location**			**0.046**
Anterior	0 (0.0%)	0 (0.0%)	
Posterior	12 (66.7%)	14 (77.8%)	
Both anterior and posterior	6 (33.3%)	2 (11.1%)	
No lesion	/	2 (11.1%)	
**Extent of lesion involvement**			**0.020**
Focal	6 (33.3%)	7 (38.9%)	
Multifocal	4 (22.2%)	5 (27.8%)	
Diffuse	8 (44.4%)	4 (22.2%)	
No lesion	/	2 (11.1%)	

Data are n (%), median (IQR). Percentages may not total 100 because of rounding. SARS-CoV-2, severe acute respiratory syndrome coronavirus 2.

## Abbreviations

COVID-19: coronavirus disease 2019
GGO: ground-glass opacity
GI: gradual improvement
MI: mismatch to improvement
PI: progression to improvement
RT-PCR: reverse transcriptase-polymerase chain reaction
SARS-CoV-2: severe acute respiratory syndrome coronavirus 2
